# Small Changes in Inter-Pulse-Intervals Can Cause Synchronized Neuronal Firing During High-Frequency Stimulations in Rat Hippocampus

**DOI:** 10.3389/fnins.2019.00036

**Published:** 2019-01-31

**Authors:** Zhouyan Feng, Weijian Ma, Zhaoxiang Wang, Chen Qiu, Hanhan Hu

**Affiliations:** Key Lab of Biomedical Engineering for Ministry of Education, College of Biomedical Engineering and Instrument Science, Zhejiang University, Hangzhou, China

**Keywords:** high-frequency stimulation, temporal patterns, population spike, synchronous firing, axonal stimulation, hippocampal CA1 region

## Abstract

Deep brain stimulation (DBS) traditionally utilizes electrical pulse sequences with a constant frequency, i.e., constant inter-pulse-interval (IPI), to treat certain brain disorders in clinic. Stimulation sequences with varying frequency have been investigated recently to improve the efficacy of existing DBS therapy and to develop new treatments. However, the effects of such sequences are inconclusive. The present study tests the hypothesis that stimulations with varying IPI can generate neuronal activity markedly different from the activity induced by stimulations with constant IPI. And, the crucial factor causing the distinction is the relative differences in IPI lengths rather than the absolute lengths of IPI nor the average lengths of IPI. In rat experiments *in vivo*, responses of neuronal populations to applied stimulation sequences were collected during stimulations with both constant IPI (control) and random IPI. The stimulations were applied in the efferent fibers antidromically (in alveus) or in the afferent fibers orthodromically (in Schaffer collaterals) of pyramidal cells, the principal cells of hippocampal CA1 region. Amplitudes and areas of population spike (PS) waveforms were used to evaluate the neuronal responses induced by different stimulation paradigms. During the periods of both antidromic and orthodromic high-frequency stimulation (HFS), the HFS with random IPI induced synchronous neuronal firing with large PS even if the lengths of random IPI were limited to a small range of 5–10 ms, corresponding to a frequency range 100–200 Hz. The large PS events did not appear during control stimulations with a constant frequency at 100, 200, or 130 Hz (i.e., the mean frequency of HFS with random IPI uniformly distributed within 5–10 ms). Presumably, nonlinear dynamics in neuronal responses to random IPI might cause the generation of synchronous firing under the situation without any long pauses in HFS sequences. The results indicate that stimulations with random IPI can generate salient impulses to brain tissues and modulate the synchronization of neuronal activity, thereby providing potential stimulation paradigms for extending DBS therapy in treating more brain diseases, such as disorders of consciousness and vegetative states.

## Introduction

Deep brain stimulation (DBS) has been developed to treat brain disorders for decades, including Parkinson’s disease, essential tremor, dystonia, epilepsy, obsessive-compulsive disorder, addiction, depression, and Alzheimer’s disease (Fridley et al., 2012; Udupa and Chen, 2015; Wichmann and DeLong, 2016; Cury et al., 2017). Despite the most successful application of the therapy for treating movement disorders such as Parkinson’s disease, DBS treatment for other diseases is not mature currently. New stimulation paradigms have been designed and tested for extending DBS therapy in treating more neurological and psychiatric disorders (Rizzone et al., 2001; Kuncel et al., 2006; Brocker et al., 2017).

Commonly, DBS utilizes biphasic pulse sequences of so-called high-frequency stimulation (HFS) with a constant pulse frequency, that is, a constant inter-pulse-interval (IPI). The efficient pulse frequency for treating movement disorders in clinic is in a range of 90–185 Hz (Rizzone et al., 2001; Kuncel et al., 2006). To improve therapy effects, irregular temporal patterns of stimulation with varying IPI or with pauses have been studied in animal experiments, computational models as well as in clinical treatments (Swan et al., 2016; Brocker et al., 2017; Cassar et al., 2017). However, the results are inconclusive. Many studies have shown that varying IPI may decrease DBS effectiveness. Even if the mean frequency of varying stimulation is as high as constant stimulation, the effectiveness of varying stimulations may be still poorer than constant stimulations (Dorval et al., 2010; Birdno et al., 2012; Kuncel et al., 2012; McConnell et al., 2016).

These studies on therapeutic effects of varying stimulation patterns have been mostly evaluated in relieving the symptoms of movement disorders (Dorval et al., 2010; Swan et al., 2016). The mechanism of effective DBS for treating these disorders has been considered to mask pathological oscillations and abnormal synchronous activity by replacing them with HFS-induced patterns of activity (Eusebio et al., 2011; Wilson et al., 2011; Medeiros and Moraes, 2014; Herrington et al., 2016). Therefore, it has been inferred that long pauses in the stimulations may fail to mask the intrinsic activity or fail to suppress the synchronous activity in the pathological brain regions, thereby decreasing the DBS effectiveness (Llinás et al., 1999; Birdno and Grill, 2008; Swan et al., 2016; McConnell et al., 2016).

However, even pauses as short as 15–25 ms inserted in a basic sequence of constant HFS at a low rate (∼2–4 Hz) are sufficient to decrease the efficacy of DBS (Birdno et al., 2007; Swan et al., 2016). It seems unlikely that such narrow and infrequent gaps could allow the target neurons to recover their intrinsic activity, because a period of seconds to minutes is needed for the neurons to return to original activity after withdrawal of constant HFS (Popovych and Tass, 2014; Feng et al., 2017). On the other hand, even a short pause (e.g., 20 ms) during constant HFS (100- or 200-Hz) can result in generation of highly synchronized population firing of neurons that differs from the asynchronous firing induced by constant HFS (Feng et al., 2014). Therefore, we propose here an alternative hypothesis for the effects of varying IPI: even if the lengths of all IPI are short enough, small changes of IPI can generate neuronal responses quite different from that induced by stimulations of constant IPI. These differences in HFS-induced activity, not a recovery of intrinsic activity, might cause different effects in DBS therapy.

To test the hypothesis, we compared the responses of neuronal populations in the hippocampal CA1 region of anesthetized rats to applied stimulation sequences with both constant IPI (control) and varying IPI in the efferent or afferent axonal fibers of pyramidal cells, the principal cells of CA1 region. The dense compact of CA1 pyramidal cells facilitates the evaluation of synchronous firing of neuronal populations by recording population spikes (PS) *in vivo*. A waveform of PS is generated from the superposition of many, simultaneous single-unit spikes surrounding the recording site (Theoret et al., 1984; Andersen et al., 2000, 2007). Furthermore, axonal activations induced by electrical pulses have been shown to play a crucial role in DBS therapy (Gradinaru et al., 2009; Hess et al., 2013; Girgis and Miller, 2016; Herrington et al., 2016). Therefore, we examined neuronal responses by directly stimulating axonal fibers. In addition, given the fact that hippocampus is a focus region of brain diseases such as epilepsy and Alzheimer’s disease (Sankar et al., 2015; Udupa and Chen, 2015), the results of the study can provide important clues for developing new stimulation paradigms to treat more brain diseases.

## Materials and Methods

### Animal Surgery and Electrode Implantation

All animal procedures used in this study conformed to the Guide for the Care and Use of Laboratory Animals (China Ministry of Health). The protocol was approved by the Institutional Animal Care and Use Committee, Zhejiang University. Twenty male Sprague-Dawley rats (adult, 310 ± 48 g) were used for *in vivo* experiments under anesthesia by urethane (1.25 g/kg, i.p.). Surgical procedures and electrode placements were similar to previous reports (Feng et al., 2013, 2017).

Briefly, one recording electrode (RE) and two stimulating electrodes (SE) were inserted into the left hippocampal region of brain. The RE was a 16-channel array (Model Poly2, Neuro-Nexus Technologies Inc., United States) and was perpendicularly positioned in the CA1 region of hippocampus. The two SE were bipolar concentric stainless-steel electrodes (Model CBCSG75, FHC Inc., United States) and were positioned in the alveus and the Schaffer collaterals of hippocampal CA1 region for antidromic and orthodromic activations of CA1 pyramidal cells, respectively. The waveforms of antidromically- and orthodromically evoked population potentials as well as signals of unit spikes appeared serially in the 16 channel recording array were used to guide the correct positioning of the electrodes.

### Recording and Stimulating

Raw signals collected in the hippocampal CA1 region were amplified 100 times by a 16-channel extracellular amplifier (Model 3600, A-M System Inc., United States) with a band-pass filtering range 0.3–5000 Hz. The amplified signals were then sampled by a PowerLab data acquisition system (Model PL3516, ADInstruments Inc., Australia) with a sampling rate of 20 kHz.

Stimulations were sequences of biphasic current pulses with each phase width of 0.1 ms and were generated by a programmable stimulator (Model 3800, A-M System Inc., United States). The current intensity of pulses was 0.3 or 0.4 mA that evoked approximately 75% maximal amplitude of PS according to an input-output curve. The curve was made by applying single pulses with a gradually increased intensity and measuring corresponding evoked PS potentials. The setting of current intensity (75% saturation value) could ensure enough activation to target region and avoid over stimulation simultaneously.

Both constant IPI and varying IPI were used. The pulse frequency of constant IPI was 100, 200, or 130 Hz. The varying IPI changed randomly in a range of 20–600 Hz (i.e., 1.67–50 ms, with a mean pulse frequency 100 Hz) or in a range of 100–200 Hz (i.e., 5–10 ms, with a mean pulse frequency ∼130 Hz).

The duration of stimulation sequences was 80, 140, or 180 s. To compare the differences between effects of random IPI and constant IPI directly, most stimulation sequences started with a 50-s period of constant IPI to reach a steady state of neuronal responses, then switched to a 10-s period random IPI and finally switched back to constant IPI for another 20 s to make a total duration 80 s. In some of the stimulations, the 60-s period (50-s constant + 10-s random IPI) was repeated twice to make a total duration of 140 s. To eliminate the possible impacts of the changes of brain state and other uncontrolled facts, in statistical evaluations, the 10-s (40 to 50 s) period of constant IPI immediately preceding the 10-s (50 to 60 s) period of random IPI was used as a control representing the “steady-state” response induced by constant IPI stimulation. Stimulation sequences with sole random IPI through a whole duration of 3 min (180 s) were also applied to show the persistent of effects induced by random IPI.

Two to four stimulation sequences with different paradigms were performed in each rat experiment. The intervals between stimulation sequences were greater than half an hour to ensure recovery from previous stimulation.

### Data Analysis

Amplitudes and areas of PS waveforms were used to evaluate the neuronal responses induced by stimulation sequences. The PS amplitude was measured as the potential difference between the negative peak of PS and the baseline before PS. The PS area was measured as the product of amplitude and half-height width of PS (Theoret et al., 1984). Additionally, “maximum amplitude” of PS within a specific period of stimulation was calculated as the average amplitude of ten largest PS waveforms to eliminate the impact of interference.

All statistical data were represented as mean ± standard deviation. “*n*” represents the number of rats for data collections or the number of stimulation sequences. Student *t*-test was used to judge the statistical significance of the differences between data groups.

In addition, to clarify the recording signals in figures, stimulation artifacts were removed by a custom-made MATLAB program with a linear interpolation algorithm. Briefly, a data segment of ∼1.0 ms around each artifact of stimulation pulse was replaced by a short line connecting the two end points of the artifact segment (Yu et al., 2016). Because the bipolar concentric stimulation electrode limited the stimulated area, the stimulation artifacts picked by the recording electrode did not induce substantial saturation in the amplifier. In addition, the detection and evaluation of PS waveforms were performed in the intervals of pulses directly on raw recording signals (0.3–5000 Hz) neither involving the removal of stimulation artifacts nor involving a low-frequency filter of field potentials.

## Results

### Responses of Neuronal Populations to Antidromic-HFS With Random Inter-Pulse-Intervals

**FIGURE 1:**
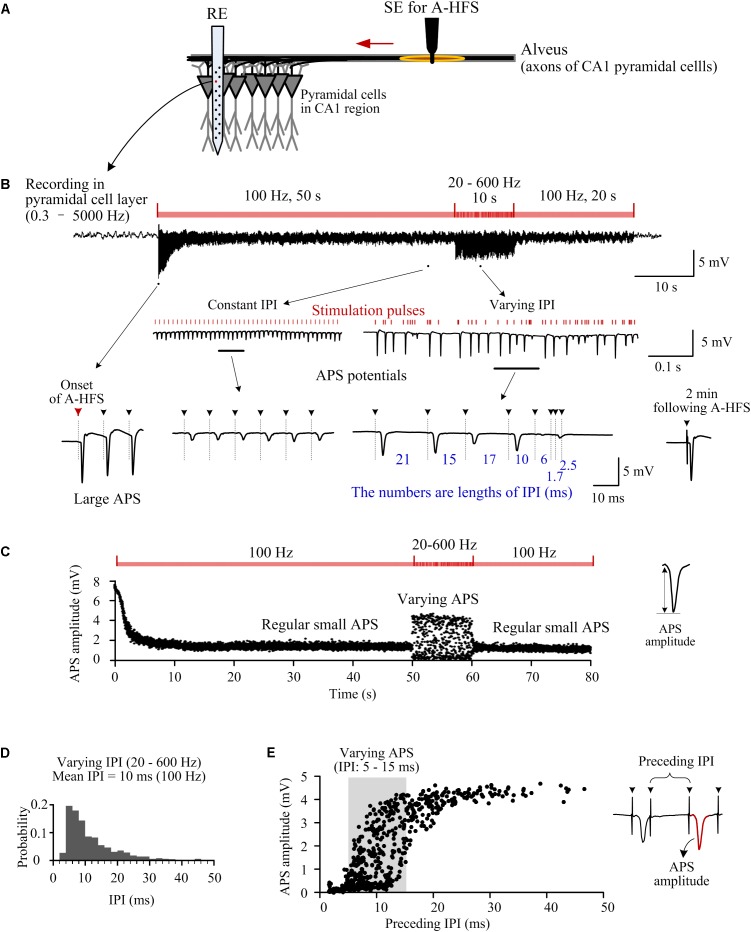
Responses of neuron populations to A-HFS with random inter-pulse-intervals (IPI). **(A)** Schematic diagram of the locations of stimulation electrode (SE) for antidromic-HFS (A-HFS) in the alveus and recording electrode array (RE) in the CA1 region. **(B)** A recording of neuronal responses to 80-s A-HFS with constant IPI (100 Hz, during 0–50 s and 60–80 s) and random IPI (20–600 Hz, during 50–60 s), together with expanded plots of APS waveforms. Red bars denote the stimulation pulses. Dashed lines with arrows denote the locations of removed artifacts of stimulation pulses. **(C)** Scatter diagrams of the amplitudes of APS evoked by each pulse during the A-HFS in **(B)**. **(D)** Probability distribution of the random IPI (20–600 Hz) for the 10-s A-HFS period in **(B)** with a mean pulse frequency of 100 Hz. **(E)** Scatter diagrams of the amplitudes of APS potentials as a function of the lengths of IPI immediately preceding the APS during the 10-s period of A-HFS with random IPI.

To compare the differences in neuronal responses to HFS with constant and random IPI in the same stimulation sequence, we utilized a HFS sequence starting with a constant pulse frequency and then switching to varying frequencies. Additionally, to focus on the reactions of neuronal axons and somata without involvement of synaptic transmissions, we firstly investigated the responses of CA1 neurons to the antidromic-HFS (A-HFS) in the efferent fibers, the alveus of hippocampal region. Figure 1 shows a typical example of results that were repeated in five individual rat experiments with the same A-HFS paradigm.

At the onset of A-HFS with a constant pulse frequency (100 Hz), each stimulation pulse evoked a large antidromically evoked population spike (APS, ∼9 mV), indicating that synchronous action potentials propagated from the efferent fibers back to the somata of the neuronal populations in the upstream region of stimulation site (Figure 1A,B). The appearance of large APS potentials formed an abrupt change at the onset of stimulation. However, the amplitudes of APS decreased rapidly in the initial period of stimulation. After tens of seconds of stimulation, the amplitudes of APS stabilized to a low level (∼20% of the initial APS amplitude, Figure 1B,C), indicating that the neuronal responses transformed from a transient phase to a steady-state phase with regular small APS evoked by each stimulation pulse. Previous studies have shown that the suppression of APS by prolonged HFS might be caused by axonal failures, a partial block of axonal activation (Jensen and Durand, 2009; Feng et al., 2013, 2014).

The small APS potentials continued with constant 100-Hz stimulation until the pulse frequency was switched into a varying pattern (20–600 Hz, mean 100 Hz) for 10 s. In this 10-s period, APS waveforms with varying amplitudes (0–4.5 mV) followed each stimulation pulse (Figure 1B,C). Afterward, when the stimulation was switched back to constant 100 Hz, regular small APS reappeared. Two minutes following the termination of the entire A-HFS, a single test pulse induced an APS waveform (in the lower right of Figure 1B) similar to that appeared at the onset of A-HFS, indicating reversibility of the stimulation effects. The recovery of neuronal responses after A-HFS was similar to previous studies with stimulations of pure constant IPI (Feng et al., 2013, 2014). To avoid redundancy, we omitted the details of recovery data.

During the 10-s A-HFS with random IPI, although the mean pulse frequency was still 100 Hz (see Figure 1D, a decline distribution was used to ensure a mean frequency of 100 Hz), the amplitudes of APS varied markedly (Figure 1E). APS amplitudes were larger (> 4 mV) with a longer preceding IPI (25–50 ms); while APS amplitudes were smaller (< 1 mV) with a shorter preceding IPI (1.7–5 ms) (note the expanded waveforms in Figure 1B and the scatter diagrams of APS amplitudes in Figure 1E). These data indicated that a longer pause of stimulation might facilitate the generation of a larger APS.

Surprisingly, however, the amplitude of evoked APS still varied in a relative large range of 0–4.5 mV with a preceding IPI in a relative short range of 5–15 ms (shadow area in Figure 1E). That is, an APS could also be large following a relative short IPI. This result implied that even if all of the lengths of IPI were short enough without longer pauses, small changes in IPI might also result in population activation of neurons with large APS that was different from the small APS induced by stimulations with constant IPI. Therefore, we next tested the hypothesis by changing the IPI in a smaller range.

### Large Population Spikes Appeared During A-HFS With Small Changes in IPI

**FIGURE 2:**
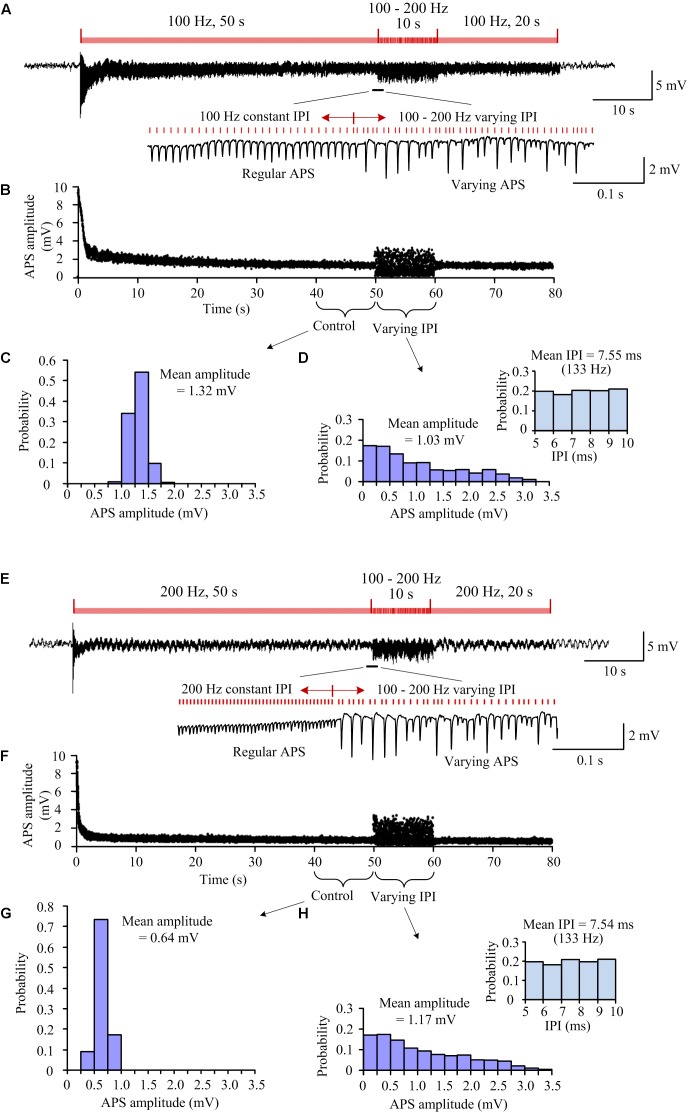
Neuronal responses to A-HFS with constant and random IPI in the range of 100–200 Hz. **(A)** A recording of 80-s A-HFS with constant IPI (100 Hz, during 0–50 s and 60–80 s) and random IPI (100–200 Hz, during 50–60 s), together with expanded plots of APS waveforms. Red bars denote the stimulation pulses. **(B)** Scatter diagrams of the amplitudes of APS evoked by each pulse during the A-HFS in **(A)**. **(C)** Probability distribution of the APS amplitudes during the 10-s control period before the stimulation of random IPI. **(D)** Probability distribution of the APS amplitudes during the 10-s period with random IPI and the probability distribution of IPI (*upper right*). **(E–H)** Corresponding plots as **(A–D)** for A-HFS with a same order of the stimulation paradigms in **(A)** but constant IPI changed to 200 Hz.

To investigate the effects of small differences in short IPI on the neuronal responses, we utilized an A-HFS sequence with random IPI only in a range of 5–10 ms (a corresponding frequency range of 100–200 Hz) following a 50-s period of constant frequency 100 or 200 Hz as a control (Figure 2).

For a 100 Hz control frequency, the mean pulse frequency (∼130 Hz) of the random-IPI uniformly distributed in 100–200 Hz was higher than the control frequency of constant 100 Hz. Furthermore, no random-IPI was greater than 10 ms (the IPI of constant 100 Hz). Nevertheless, during the period of random IPI, some of the pulses induced larger APS while some other pulses induced no APS (Figure 2A,B). To compare the neuronal responses to constant and random IPI, the distributions of APS amplitudes within two neighboring periods of 10-s stimulation were evaluated: 40–50 s of the A-HFS with constant 10 ms (Figure 2C) and 50–60 s of the A-HFS with random IPI (Figure 2D). The probability distribution of APS amplitudes during constant IPI was approximate to a normal distribution with a small range 0.83–1.90 mV and a mean amplitude 1.32 mV, while the probability distribution of APS amplitudes during random IPI was a decline distribution with a large range 0–3.22 mV and a mean amplitude 1.03 mV. Additionally, the decline distribution of APS amplitudes induced by random IPI was different from the uniform distribution of random IPI (in the upper right of Figure 2D), indicating a nonlinear relationship between the neuronal responses and the lengths of IPI.

Similar results were observed with a same stimulation paradigm but an increased control frequency of 200 Hz (Figure 2E–H). The increase in control frequency resulted in a decrease of the steady-state APS amplitude to a mean value 0.64 mV (with a smaller range 0.36–1.15 mV) during the 40–50 s of the A-HFS (Figure 2G). However, during the 50–60 s of the A-HFS when the stimulation was switched into the pattern of random IPI (still 100–200 Hz), the change of APS amplitudes again increased to a larger range 0–3.38 mV with a mean value 1.17 mV (Figure 2H), which were similar to the situation with a lower control frequency 100 Hz (Figure 2D).

**FIGURE 3:**
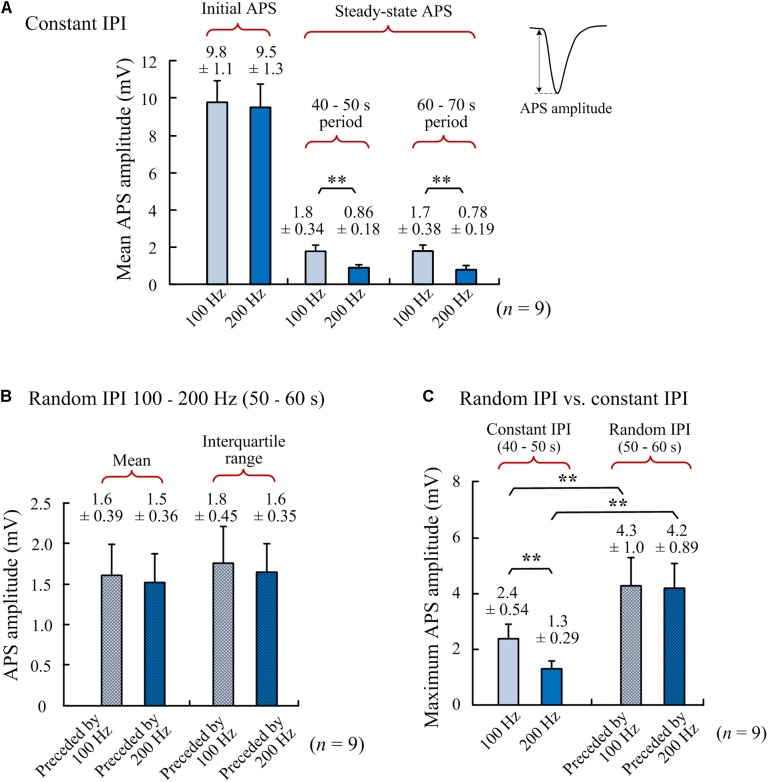
Comparisons of APS amplitudes among different periods of A-HFS. **(A)** Amplitudes of the initial APS (evoked by the very first pulse at the onset of A-HFS) and the steady-state APS (mean amplitudes in 40–50 s and 60–70 s of stimulations) during A-HFS with constant IPI 100 and 200 Hz, respectively. **(B)** Mean APS amplitudes and interquartile ranges of APS amplitudes during 10-s A-HFS periods (50–60 s) with random IPI (100–200 Hz) preceded by 100- or 200-Hz constant stimulations, respectively. **(C)** Maximum APS amplitudes during A-HFS periods with constant IPI (steady-state) and with random IPI. ^∗∗^*P* < 0.01, *n* = 9, *t*-test.

The above stimulations (Figure 2) with both 100 and 200 Hz as a control frequency were repeated in nine rats. Statistical data of the nine experiments showed that with a similar initial APS amplitude induced by the very first pulse at the onset of A-HFS (Figure 3A), the mean steady-state APS amplitude of 200-Hz A-HFS was significantly smaller than the corresponding value of 100-Hz A-HFS during control periods of both 40–50 s and 60–70 s. This result was consistent with previous reports, indicating that constant A-HFS with a higher frequency can suppress APS more by inducing deeper failures in axonal conduction (Jensen and Durand, 2009; Feng et al., 2013, 2014). However, both the mean and the interquartile range of APS amplitudes during the 10-s periods of random IPI inserted in 200-Hz control A-HFS were not significantly different from the values with 100-Hz control A-HFS (Figure 3B). This result indicated that the neuronal responses to random IPI were not correlated with the preceding suppression level of APS.

More interestingly, despite the limited range of random IPI in 5–10 ms (200–100 Hz), the amplitude ranges of varying APS induced by the random IPI were far beyond the steady-state amplitudes of APS induced by constant IPI of 100 or 200 Hz. The maximum APS amplitudes induced in the periods of random IPI were significantly greater than the maximum APS amplitudes induced in the preceding control periods of both 100- and 200-Hz A-HFS (Figure 3C). Additionally, some pulses of random IPI failed to induce APS (amplitude = 0) whereas each pulse of constant IPI induced APS (Figure 2).

These results showed that although the range of random IPI was limited in the small range of 100 to 200 Hz, the APS amplitudes induced by random IPI were not limited between the amplitude levels of the steady-state APS induced by constant 100 and 200 Hz. Because larger APS represents higher synchronization of action potential firing of neuronal populations, we next tested the hypothesis that irregularity of IPI could facilitate synchronous firing of neurons by reshaping firing timing without significantly changing the total amount of neuronal firing.

### Reshaping the Neuronal Firing Timing by A-HFS With Random IPI

**FIGURE 4:**
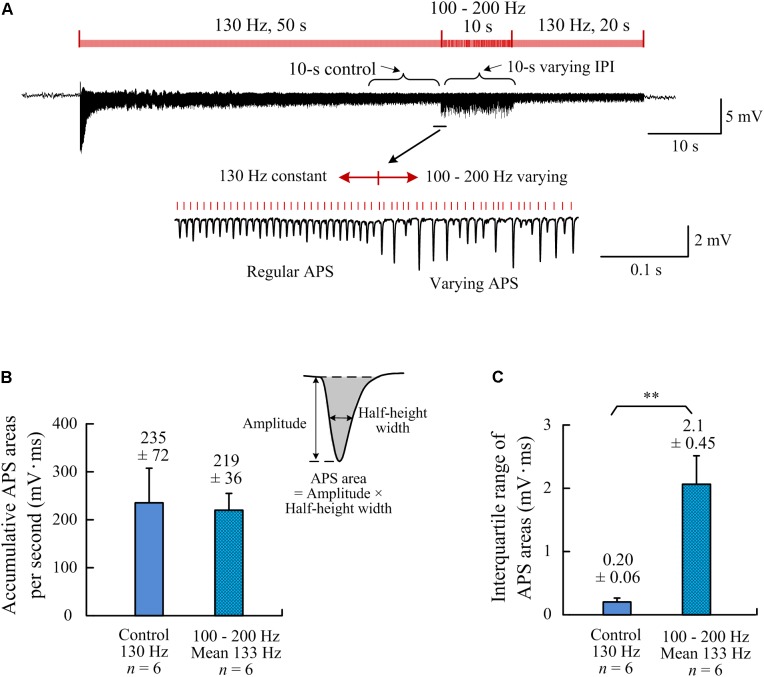
Comparisons of neuronal firing amounts quantified by APS areas during the two different A-HFS periods with constant IPI (control) and with random IPI (but with same amount of pulses). **(A)** a recording of 80-s A-HFS with constant IPI (130 Hz, during 0–50 s and 60–80 s) and random IPI (100–200 Hz, mean ∼130 Hz, during 50–60 s), together with expanded plots of APS waveforms. Red bars denote the stimulation pulses. **(B)** Comparisons of the accumulative APS areas between the control period of constant IPI and the period of random IPI (*P* = 0.64, *n* = 6, *t*-test). **(C)** Comparisons of the interquartile ranges of APS areas during the two different periods (^∗∗^*P* < 0.01, *n* = 6, *t*-test).

Area of an APS waveform can be used to represent the number of neurons that fire action potential synchronously to form the APS (Theoret et al., 1984). Therefore, we used the index of accumulative APS areas to compare the amounts of neuronal firing between the stimulation periods with constant and random IPI. To evaluate the neuronal firing under same amount of stimulation pulses (i.e., same amount of electrical charge injected), the frequency of control stimulation was set at 130 Hz, similar to the mean frequency (133 Hz) of the stimulation with random IPI uniformly distributed in the range 100–200 Hz. The accumulative APS areas per second were similar during the two 10-s periods: the control periods of constant IPI and the period of random IPI (Figure 4A,B; *P* = 0.64, *n* = 6, *t*-test). Nevertheless, the interquartile range of APS areas during random IPI was significantly greater than that during constant IPI (Figure 4C; *P* < 0.01, *n* = 6, *t*-test). These data indicated that the differences of IPI caused a redistribution of the neuronal firing without significantly altering the total amount of neuronal firing.

**FIGURE 5:**
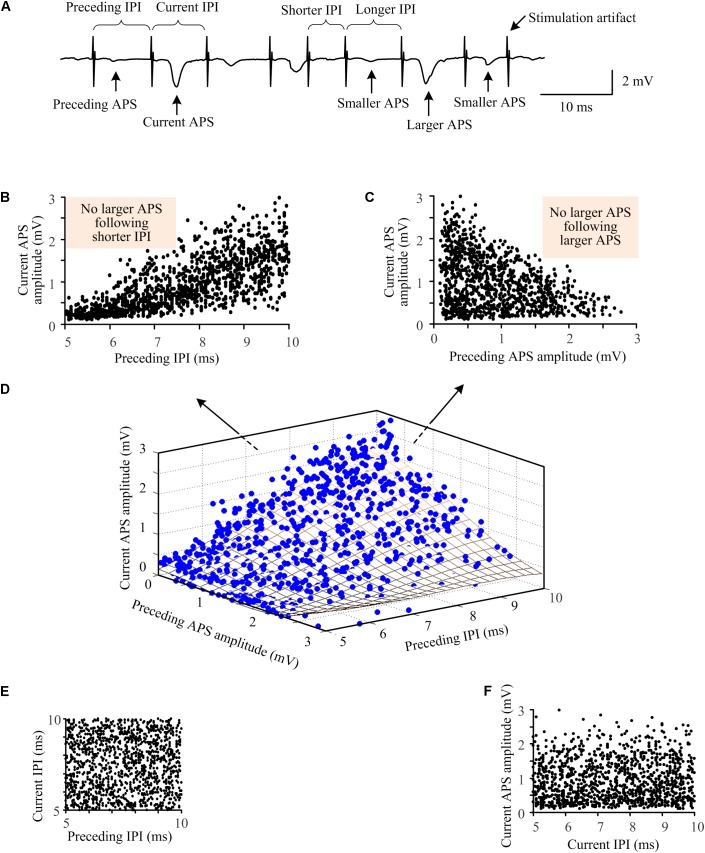
Relationships among the amplitudes of neighboring APS waveforms and the lengths of IPI during A-HFS with random IPI. **(A)** A segment of signal illustrating the definitions of indexes. **(B)** The amplitudes of current APS as a function of the lengths of preceding IPI. **(C)** The amplitudes of current APS as a function of the amplitudes of preceding APS. **(D)** Three-dimensional plot of the amplitudes of current APS as a function of both the amplitudes of preceding APS and the lengths of preceding IPI. The fitting surface (grid surface) denotes the distribution trend of the relationship. **(E)** The length of current IPI did not correlate with the length of preceding IPI, resulting in a uniform distribution between neighboring IPI. **(F)** The amplitude of current APS did not correlate with the length of current IPI.

To further investigate the relationships between the synchronization of neuronal firing and the random IPI, we examined the correlations among the amplitude of current APS, the amplitude of preceding APS, and the length of preceding IPI (Figure 5A). During the 10-s A-HFS with random IPI in the small range of 5–10 ms, larger APS (> 1.8 mV) only followed longer IPI but not shorter IPI (5–7.5 ms, the shade area in Figure 5B). Although smaller APS also appeared following longer IPI, many of those smaller APS had a larger preceding APS (Figure 5C,D). Additionally, although two longer IPI could exist next to each other in the uniform distribution of random IPI (Figure 5E), two larger APS never appeared consecutively (see the shade area in Figure 5C). This indicated that a larger preceding APS could prevent a second larger APS immediately induced by the next pulse. As expected, the APS amplitudes did not correlate with current IPI since APS was induced by the preceding pulse but not by the succeeding one (Figure 5F). Similar results were obtained in all of the twenty rat experiments by applying A-HFS with the random IPI.

**FIGURE 6:**
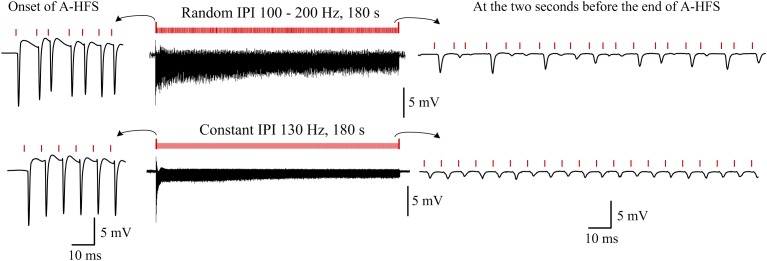
Examples of typical responses of neuron populations to a 180-s A-HFS sequence with entire random IPI in a frequency range of 100–200 Hz (*upper row*) and to a 180-s A-HFS sequence with constant IPI of 130 Hz frequency (*bottom row*). Induced potentials at the onset of A-HFS and at seconds before the end of A-HFS are enlarged. Red bars denote the stimulation pulses.

Additionally, to demonstrate the persistent of effects induced by random IPI, in five rat experiments, a stimulation sequence with random IPI (100–200 Hz) through a whole duration of 3 min (180 s) was applied (Figure 6 upper row). In these same experiments, a control of 3-min stimulation with a constant IPI of 130 Hz frequency (Figure 6 bottom row) was also applied. The APS events induced at the onsets of the two stimulation sequences were similar. However, during the late periods of stimulations, corresponding to the periods that steady small APS events were induced during A-HFS with constant IPI, large APS appeared irregularly during A-HFS with random IPI. Except the first few seconds of the stimulations, the differences of neuronal responses persisted through the remaining ∼3 min periods of the two separate stimulations. The results indicated that the distinct neuronal responses induced by small changes in IPI (5–10 ms) could last steadily, not transiently.

**FIGURE 7:**
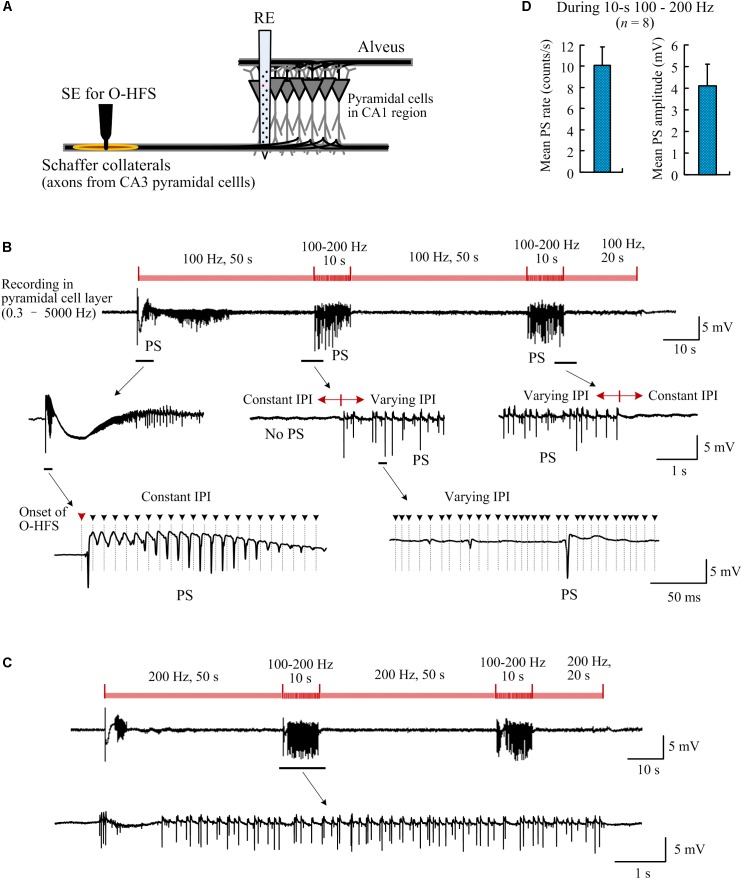
Population spikes induced by orthodromic-HFS (O-HFS) with random IPI. **(A)** Schematic diagram of the locations of stimulation electrode (SE) for O-HFS in the Schaffer collaterals and recording electrode array (RE) in the hippocampal CA1 region. **(B)** A recording example of neuronal responses to 140-s O-HFS with constant IPI (100 Hz, during 0–50 s, 60–110 s, and 120–140 s) and two inserted periods of random IPI (100–200 Hz, during 50–60 s, and 110–120 s). Expanded plots show the orthodromically evoked population spikes (PS). Dashed lines with arrows denote the locations of removed stimulation artifacts. **(C)** A recording example of neuronal responses to a similar stimulation order as in **(B)** but with constant IPI increased to 200 Hz. **(D)** The mean PS rate and the mean PS amplitude during the two periods of 10-s random IPI in both **(B)** and **(C)**, *n* = 8 stimulation sequences in four rats.

The above results were all obtained from antidromic stimulations without involving synaptic transmissions. They indicated that during axonal antidromic-HFS, despite the high enough mean-frequency of stimulation, small differences in IPI may significantly change the firing time of neurons to facilitate the generation of highly synchronized action potentials in upstream neuronal somata. Because the stimulation-induced activation of axons can conduct in both antidromic and orthodromic directions simultaneously (Udupa and Chen, 2015; Feng et al., 2017), we hypothesized that the same stimulation paradigms with random IPI applied orthodromically at the afferent fibers of CA1 region could also induce irregular population activity in the post-synaptic neurons downstream. Thus, we next tested this hypothesis by applying orthodromic-HFS (O-HFS) at the Schaffer collaterals of hippocampal CA1 region (Figure 7A).

### Synchronous Firing Induced by Orthodromic-HFS With Random IPI

To compare the neuronal responses to O-HFS with constant IPI and random IPI, a period of 50-s O-HFS with constant IPI (10 ms, 100 Hz) was firstly applied and then it was switched to 10-s O-HFS with random IPI (5–10 ms, 200–100 Hz). The identical stimulation paradigm repeated twice and was finally followed by a 20-s constant IPI to complete a total of 140-s stimulation. Consistent with previous reports (Feng et al., 2013, 2017), at the onset of O-HFS, the first pulse evoked a large orthodromically evoked population spike (OPS) followed by a period with PS potentials (Figure 7B). After seconds of continuous stimulation of 100 Hz, in the steady-state of neuronal responses, OPS activity disappeared. Nevertheless, in this steady-state period without OPS events, unit activity increased (see reference Feng et al., 2017 for details, similar data obtained in the present study are omitted here).

Once the stimulation was switched to O-HFS with random IPI (5–10 ms), OPS activity reappeared. Afterward, immediately following the stimulation switched back to constant IPI (10 ms), OPS disappeared again. The reappearance of OPS was repeated in the second turn of stimulation with random IPI (Figure 7B).

Changing the constant IPI from 100 to 200 Hz (5 ms IPI) and keeping the other parameters in the stimulation sequence unchanged, OPS activity also appeared during the two inserted periods with random IPI in the same range of 5–10 ms. No OPS appeared during the steady-state periods with 200-Hz constant IPI except the initial transient-period of O-HFS (Figure 7C). In four rat experiments applied by two stimulations with a 100 and 200 Hz control frequency separately (total 8 stimulation sequences), during the two periods of 10-s random IPI, the mean OPS rate was 10.1 ± 1.7 counts/s and the mean OPS amplitude was 4.1 ± 1.0 mV (*n* = 8 stimulation sequences in four rats; Figure 7D). The OPS waveforms were detected by a threshold of 0.5 mV.

These results indicated that during prolonged O-HFS at afferent fibers, pulses with random IPI could irregularly induce synchronized firing of action potentials in the neuronal populations downstream, while pulses with constant IPI did not.

## Discussion

The major findings of the present study are: (1) During antidromic stimulations of efferent axons (without involving synaptic transmissions), random IPIs in a high-frequency range of 100–200 Hz can generate highly synchronized firing in the neuronal somata, whereas stimulations with constant IPI cannot. (2) The synchronous firing may be generated by reshaping the timing of neuronal firing with the small changes of IPI rather than by increasing the amount of neuronal firing. (3) Similar to antidromic stimulations, during orthodromic stimulations of afferent axons, HFS with random IPI can also induce synchronous activity in downstream neurons through monosynaptic transmission. The possible mechanisms underlying these observations are discussed below.

### Small Relative Differences in IPI Lengths Can Alter Neuronal Activity Markedly

The novel finding of our present study is that even if without long pauses, small changes in short enough IPI corresponding to HFS over 100 Hz can generate a pattern of neuronal activity markedly different from the activity induced by HFS of constant IPI. One could assume that the generation of highly synchronous firing of neurons by random IPI might be attributed to the fact that some of the lengths in random IPI were longer than constant IPI of control stimulation. However, even when all of the IPI (within 5–10 ms) of random stimulation were not longer than the constant IPI (e.g., 10 ms) of control stimulation (Figure 2A–C, 7B), the random stimulation with a higher mean frequency (∼130 Hz) could still induce more synchronous neuronal firing than constant IPI with a lower frequency (100 Hz). The results clearly showed that the crucial stimulation parameter facilitating the generation of highly synchronized firing of neurons (i.e., the larger PS) is the relative differences in IPI lengths rather than the absolute lengths of IPI nor the average lengths of IPI. To our knowledge, this is a novel finding that has not been reported before.

The importance of temporal pattern of stimulation on DBS efficacy, not simply stimulation frequency, has been recognized in many reports (Montgomery, 2005; Baker et al., 2011; Hess et al., 2013). Previous studies have shown that DBS with varying IPI is less effective than with constant IPI (Dorval et al., 2010; Birdno et al., 2012). However, most of these studies have focused on the effects of long pauses that are at least longer than constant IPI of control stimulations (Birdno et al., 2012; Kuncel et al., 2012; McConnell et al., 2016). Long pauses could decrease the HFS effects of DBS in masking or suppressing the synchronous activity of pathological neurons in some disorders (Llinás et al., 1999; Birdno and Grill, 2008; McConnell et al., 2016; Swan et al., 2016). Nevertheless, the present study provides new clues to explain the efficacy decrease of DBS with varying IPI. That is, HFS with varying IPI could induce synchronous activity in target neurons rather than suppress synchronous activity. In addition, long pauses are not necessary to induce the synchronous activity. It might explain why even pauses far shorter than pathological oscillations may destroy the DBS efficacy (Birdno et al., 2007; Swan et al., 2016). Nonlinear dynamics in neuronal responses to varying IPI might be an underlying mechanism.

### Random IPI May Induce Synchronous Firing Through a Nonlinear Recovery Course of HFS-Induced Failures

During steady-state periods of axonal A-HFS with constant IPI, the antidromically evoked population spikes (APS) were suppressed (Figure 1, 2, 3A). Because the APS potentials are induced by the stimulation excitations traveling along axons antidromically to cell bodies, not involving synaptic transmission; only failures in axons or/and cell bodies can result in the APS suppression. Previous studies have shown that HFS-induced axonal failures may cause the APS decrease by preventing the stimulated axons from generating action potentials following every stimulation pulse (Jensen and Durand, 2009; Zheng et al., 2011; Feng et al., 2013; Rosenbaum et al., 2014). The axonal failures may be caused by potassium accumulation in the narrow space immediately outside the axon membrane that results in a depolarization block (Poolos et al., 1987; Shin et al., 2007; Bellinger et al., 2008). Additionally, the axonal block may be intermittent or partial (Jensen and Durand, 2009; Feng et al., 2017; Guo et al., 2018). That is, the blocked axons could recover in turn and fire an action potential every several pulses thereby generating small APS following each pulse (Figure 2, 3A). Furthermore, the extent of axonal block depends on stimulation frequency. With a higher stimulation frequency, fewer axons can follow each stimulation pulse to generate an action potential (Feng et al., 2013, 2014; Guo et al., 2018). Thus, the APS amplitudes during steady-state periods of 200-Hz A-HFS were smaller than the values during 100-Hz A-HFS (Figure 3A).

This frequency-dependent block of axons implies that the amplitudes of evoked APS could be positively correlated to the length of preceding IPI. That is, if the IPI varies in a range of 5–10 ms, the APS amplitudes should be expected to vary in a range limited by the upper and lower limits of APS amplitudes induced by constant 100- and 200-Hz A-HFS, respectively. Surprisingly, our present study shows that the maximum APS amplitudes induced by random IPI were significantly larger than the limit values of corresponding A-HFS with constant IPI (Figure 3C).

In addition, for a similar mean frequency (i.e., same amount of stimulation pulses), the total amount of neuronal firing induced by random IPI was similar to that induced by constant IPI (Figure 4B). Therefore, randomization of IPI could only redistribute the firing time of neurons but not increase their firing amount. Presumably, a nonlinear time-course of recovery from HFS-induced axonal block could cause the redistribution of firing time by random IPI. The nonlinearity of recovery may be due to highly nonlinear dynamics of ionic-channel activations in cell membranes and their response to stimulations (Hodgkin and Huxley, 1952; Grill, 2015).

One can imagine that with constant IPI, every time a stimulation pulse arrives, a similar number of blocked axons would have recovered readily to respond to the pulse thereby generating APS with uniform and small amplitudes. In contrast, with random IPI, the number of ready axons for each coming pulse would be different due to multiple factors, such as the length of preceding IPI and the history of axon firing (Figure 5), that are determined by the nonlinear dynamics of axonal membranes. A pulse arriving “ahead of time”, i.e., a relatively shorter IPI, could prevent some axons from firing and postpone their firing to follow the second incoming pulse, together with the firing of other available axons, thereby forming a larger APS. Because of the extension of refractory period of axons by HFS-induced depolarization block (Feng et al., 2014), the larger APS might prevent the third incoming pulse from inducing axonal firing, causing no APS following the third pulse. Thus, the random appearances of “extreme” large and “extreme” small APS would result in the large range of APS amplitudes (see Figure 2–5).

The APS potentials induced by A-HFS do not involve synaptic transmission. During O-HFS, the axonal excitations travel orthodromically to terminals and then through synapses to post-synaptic neurons. The additional effect of synaptic transmission in the orthodromic responses of neurons might further increase the timing variability in neuronal firing among random IPI thereby generating large population spikes in the post-synaptic neurons of downstream regions (Figure 7).

Taken together, a nonlinear recovery course of HFS-induced axonal block could be responsible substantially for producing highly synchronized firing during stimulation of random IPI even with a high enough mean frequency. The different neuronal responses were switched back and forth in seconds immediately following the switches between random IPI and constant IPI in the antidromic-HFS without involving synapses. Therefore, the changes of neuronal responses could hardly be caused by hippocampal plasticity that mainly generates in synapses and is characterized by long-term changes. Nevertheless, nonlinear dynamics in neuronal elements other than axons, such as cell bodies and synaptic transmissions might also contribute to the synchronous activity induced by varying IPI and await further studies.

### Implication and Limitation

The distinct pattern of neuronal responses to the stimulations with random IPI provides clues for extending the DBS therapy to brain diseases other than movement disorders.

Previous studies have shown that conventional DBS with a constant pulse frequency can regularize neuronal firing patterns. The regularization of neuronal firing may be crucial for the clinical effectiveness of DBS in treating movement disorders (Kuncel et al., 2007; Birdno and Grill, 2008; Dorval et al., 2008). However, different brain diseases are caused by distinct pathological mechanisms thereby likely requiring different DBS patterns to obtain desirable efficacy. For example, DBS has been used to treat disorders of consciousness caused by severe traumatic brain injury to arouse patients from minimally conscious state (Schiff et al., 2007). Studies have shown that stimulations with random IPI might be more effective for increasing arousal than conventional constant IPI (Quinkert and Pfaff, 2012; Tabansky et al., 2014). The present study suggests that synchronous activation may be induced by random IPI, generating salient impulses to brain tissues. It seems reasonable to speculate that the impulses may arouse an “inactive” brain more effectively than regular and mild inputs from constant IPI. Therefore, the irregular stimulation could be potential paradigms for advancing DBS therapy in treating more brain diseases, such as disorders of consciousness and memory decline (Schiff et al., 2007; Laxton et al., 2010).

Taking the advantage of the dense and lamellar distributions of hippocampal neurons to facilitate the evaluation of synchronization of neuronal firing, we performed the present study in the hippocampal region. Besides direct revelations for treating diseases generated in hippocampi such as epilepsy and Alzheimer’s disease (Sankar et al., 2015; Udupa and Chen, 2015), the results of direct responses of hippocampal neurons to stimulations with random IPI may also extend to neurons in other brain regions based on general properties of most brain neurons. Nevertheless, brain regions other than hippocampus need to be investigated to finally verify the universality of the neuronal responses to stimulations of random IPI.

Although urethane was used in the *in vivo* experiments here, the influences of the anesthetic on neuronal activity in brain are slight (Shirasaka and Wasterlain, 1995; Sceniak and Maciver, 2006). Additionally, we used a control of constant IPI in the same stimulation sequence with random IPI. Therefore, a small potential decrease of background neuronal activity by the use of anesthetic should not affect the comparison of neuronal responses to the stimulations switched back and forth in seconds between constant IPI and random IPI. Nevertheless, further studies are needed to duplicate the results in awake animals. Finally, the therapeutic efficacies of random IPI await investigations in pathological models of animals other than normal animals.

## Conclusion

Previous studies have shown that high-frequency pulse stimulation with constant IPI can desynchronize neuronal activity or generate asynchronous firing in target neurons (Medeiros and Moraes, 2014; Popovych and Tass, 2014; Feng et al., 2017), whereas the present study shows that small random changes of IPI can result in synchronous firing of population neurons even without long IPI. The results suggest that small changes of IPI can modulate the synchronization of neuronal activity during HFS. The novel finding provides clues for extending the DBS therapy widely to more brain diseases.

## Author Contributions

ZF and ZW designed the experiments and interpreted the data. WM, CQ, HH, and ZW performed the experiments and analyzed the data. ZF wrote the manuscript. All authors approved the final version of the manuscript to be published and agreed to be accountable for all aspects of the manuscript.

## Conflict of Interest Statement

The authors declare that the research was conducted in the absence of any commercial or financial relationships that could be construed as a potential conflict of interest.
